# Characterization data of reference cement CEM I 42.5 R used for priority program DFG SPP 2005 “Opus Fluidum Futurum – Rheology of reactive, multiscale, multiphase construction materials”

**DOI:** 10.1016/j.dib.2019.104699

**Published:** 2019-10-22

**Authors:** Z.C. Lu, M. Haist, D. Ivanov, C. Jakob, D. Jansen, S. Leinitz, J. Link, V. Mechtcherine, J. Neubauer, J. Plank, W. Schmidt, C. Schilde, C. Schröfl, T. Sowoidnich, D. Stephan

**Affiliations:** a Department of Civil Engineering, Technische Universität Berlin, 13355, Berlin, Germany; b Institute of Building Materials, Leibniz Universität Hannover, 30167, Hannover, Germany; c Institute of Concrete Structures and Building Materials (IMB) and Materials Testing and Research Institute (MPA Karlsruhe), Karlsruher Institue für Technologie, 76131, Karlsruhe, Germany; d Institute for Particle Technology, Technische Universität Braunschweig, 38106, Braunschweig, Germany; e GeoZentrum Nordbayern, Mineralogy, Friedrich-Alexander Universität Erlangen-Nürnberg, 91054, Erlangen, Germany; f Bundesanstalt für Materialforschung und - Prüfung (BAM), 12205, Berlin, Germany; g Institute of Construction Materials, Technische Universität Dresden, 01159, Dresden, Germany; h Department of Chemistry, Technische Universität München, 85748, Garching, Germany; i F.A. Finger-Institute for Building Materials, Bauhaus-Universität Weimar, 99421, Weimar, Germany

**Keywords:** Portland cement, Characterization, DFG SPP 2005

## Abstract

A thorough characterization of starting materials is the precondition for further research, especially for cement, which contains various phases and presents quite a complex material for fundamental scientific investigation. In the paper at hand, the characterization data of the reference cement CEM I 42.5 R used within the priority program 2005 of the German Research Foundation (DFG SPP 2005) are presented from the aspects of chemical and mineralogical compositions as well as physical and chemical properties. The data were collected based on tests conducted by nine research groups involved in this cooperative program. For all data received, the mean values and the corresponding errors were calculated. The results shall be used for the ongoing research within the priority program.

**Table:** Specifications Table

Subject	Ceramics and Composites
Specific subject area	Building materials; Cement
Type of data	Table; Image; Graph; Figure
How data was acquired	XRD; SEM; EN 196-1: 2016; EN 196-2: 2013; EN 196-3: 2016; EN 196-6: 2018; EN 196-11: 2018; EN 1097-7: 2008; ISO 13320: 2009; ISO 9277: 2010
Data format	Raw; Analyzed
Parameters for data collection	Chemical composition; Phase contents; Density; Specific surface area; Particle size; Calorimetry; Water demand; Setting time; Mechanical strength
Description of data collection	Firstly a thorough characterization on CEM I 42.5 R was made by in total 9 research groups. Then the data were collected and compared. Furthermore, the mean values and the corresponding errors were calculated based on the collective data.
Data source location	Seven universities, one research institute, and one company as shown in Table 1
Data accessibility	Repository name: Deposit Once Data identification number: https://doi.org/10.14279/depositonce-9023 Direct URL to data: https://depositonce.tu-berlin.de/handle/11303/10032
Related research article	The data presented here will be cited by the upcoming research publications financed by DFG SPP 2005

**Table:** 

**Value of the Data** • The data are useful because a well characterization on CEM I 42.5 R from aspects of composition and properties are shown in this paper. Besides, the corresponding variation trend on cementitious materials is also included. • All the research groups involved in the DFG SPP 2005 priority program and other related researchers can use these data for their further study. • The data provide a solid foundation for the further research involved in the DFG SPP 2005 priority program. Besides, all researchers can refer to this variation trend on cementitious materials in their own study. • Seven universities, one research institute and one company are involved to conduct common characterization tests on the same samples.

## 1 Data

**Fig. 1:**
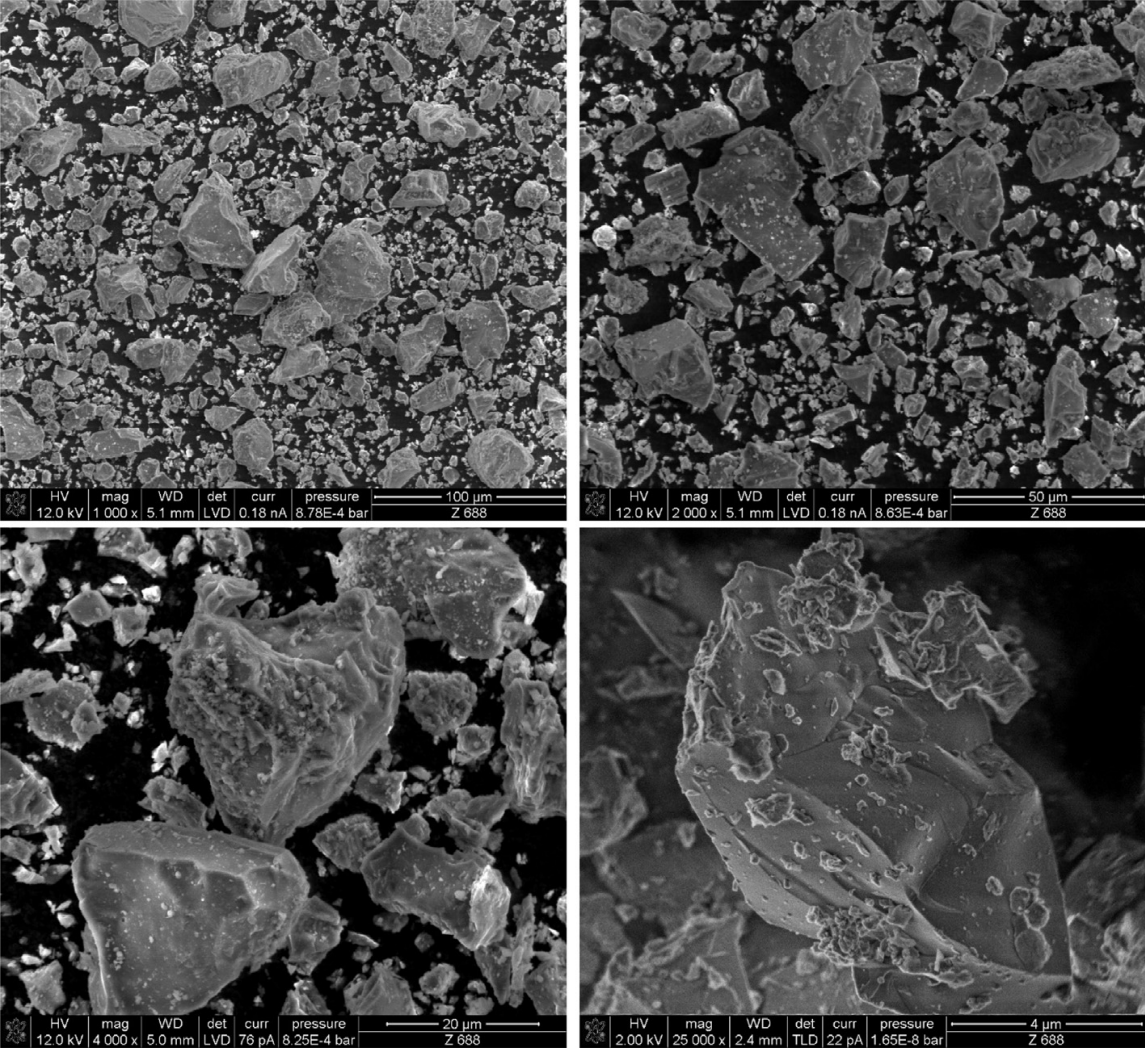
SEM pictures of CEM I 42.5 R with different magnifications.

Table 1 lists the universities, research institute, and cement company involved in the characterization of the CEM I 42.5 R and the abbreviations are explained respectively. Fig. 1 shows selected SEM pictures of cement grains with different magnifications.

**Table 1:** Universities, research institute and the company involved in the characterization.

Acronym	Affiliation
BAM	Bundesanstalt für Materialforschung und -prüfung
BUW	Bauhaus-Universität Weimar
FAU	Friedrich-Alexander Universität Erlangen-Nürnberg
Heidelberg	HeidelbergCement AG
KIT	Karlsruher Institut für Technologie
TUB	Technische Universität Berlin
TUBS	Technische Universität Braunschweig
TUDD	Technische Universität Dresden
TUM	Technische Universität München

### 1.1 Characterization data of oxide composition and phase contents

**Fig. 2:**
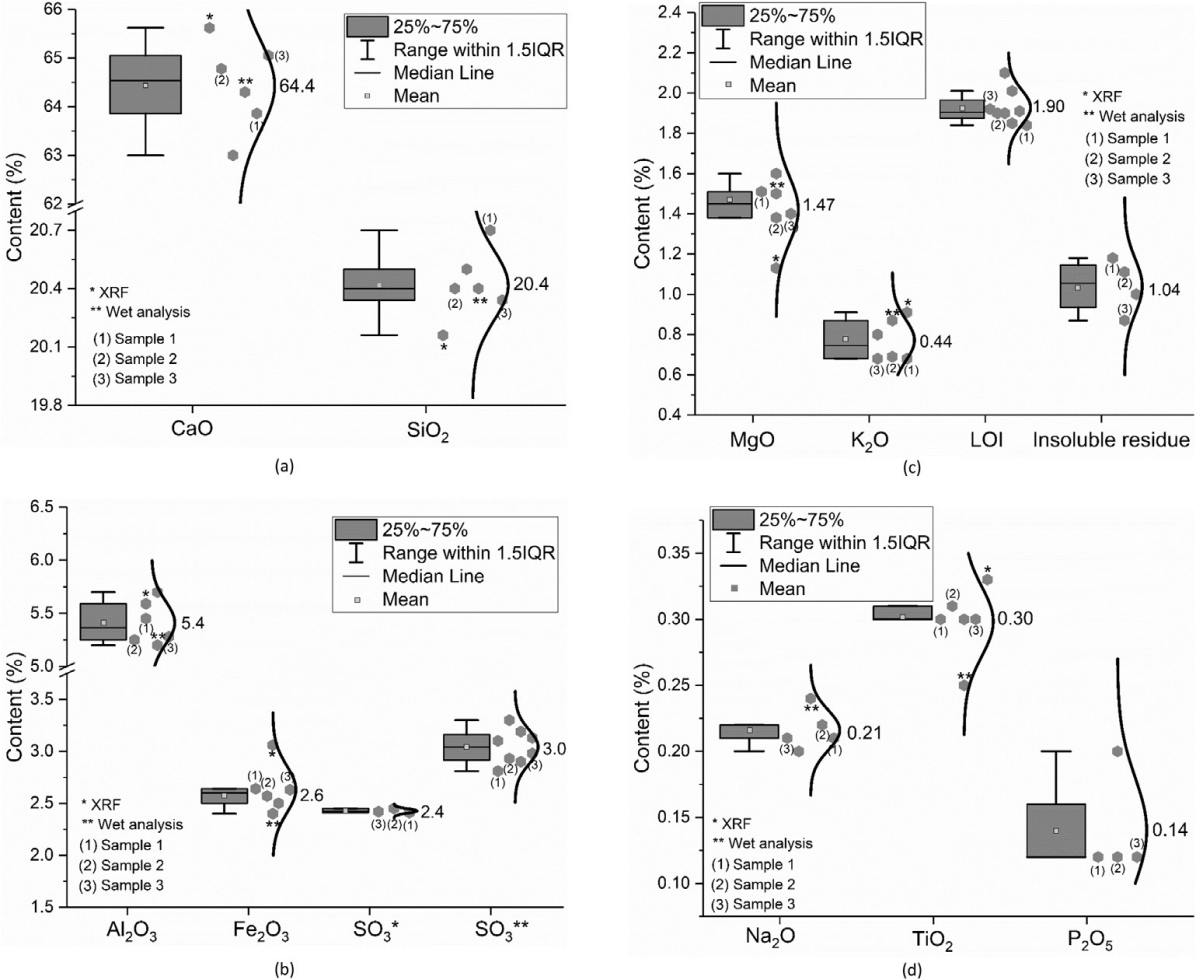
Oxide composition of CEM I 42.5 R; (a) CaO and SiO_2_; (b) Al_2_O_3_, Fe_2_O_3_ and SO_3_; (c) MgO, K_2_O, loss on ignition and insoluble residue; (d) Na_2_O, TiO_2_ and P_2_O_5_.

In Fig. 2 the oxide composition (CaO, SiO_2_, Al_2_O_3_, Fe_2_O_3_, SO_3_, MgO, K_2_O, Na_2_O, TiO_2_ and P_2_O_5_), insoluble residue as well as the loss on ignition (LOI) of CEM I 42.5 R measured by the different participating groups according to EN 196-2: 2013 [1] are shown. It should be mentioned that the data denominated as (1) to (3) were measured by one research group from one single batch but different bags. In Fig. 2(b) SO_3_* means the value obtained by the X-ray fluorescence analysis (XRF) and SO_3_** indicates the value captured by the wet chemistry method. The same meanings of * and ** are also suitable for the other data shown in Fig. 2. Unless otherwise stated, the oxide composition shown in Fig. 2 is measured based on XRF analysis. Furthermore, due to the quite low content of Cl^−^ of 0.02 wt.% only, the amount of Cl^−^ is not included in Fig. 2.

In the legend of the figures of this paper, IQR means the interquartile range, namely the range between 25^th^ and 75^th^ percentiles (as shown in the area in the grey box). The specific explanation could be found on the website [2]. The error bar shows the range within 1.5 times of IQR. The median line indicates the 50^th^ percentile and the mean value is calculate based on data from all the samples within the 1.5 IQR range and does not include outliers.

**Fig. 3:**
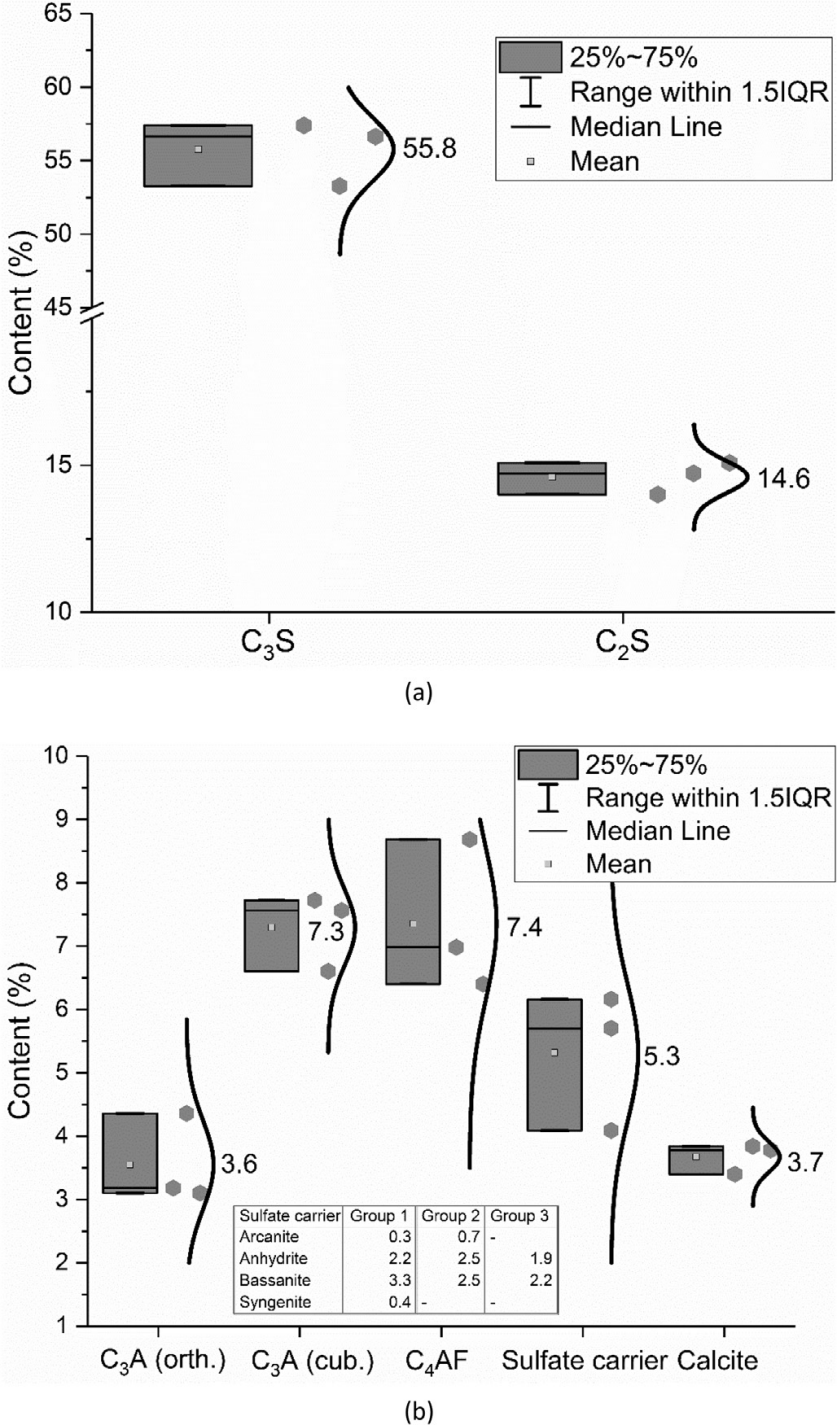
Phase contents in CEM I 42.5 R; (a) C_3_S and C_2_S; (b) C_3_A, C_4_AF, sulfate carrier and calcite.

Fig. 3 shows the phase contents of CEM I 42.5 R based on the results from three different groups through the method of powder-XRD combined with quantification of the patterns according to the Rietveld refinement method [3].

### 1.2 Characterization data of physical properties

**Fig. 4:**
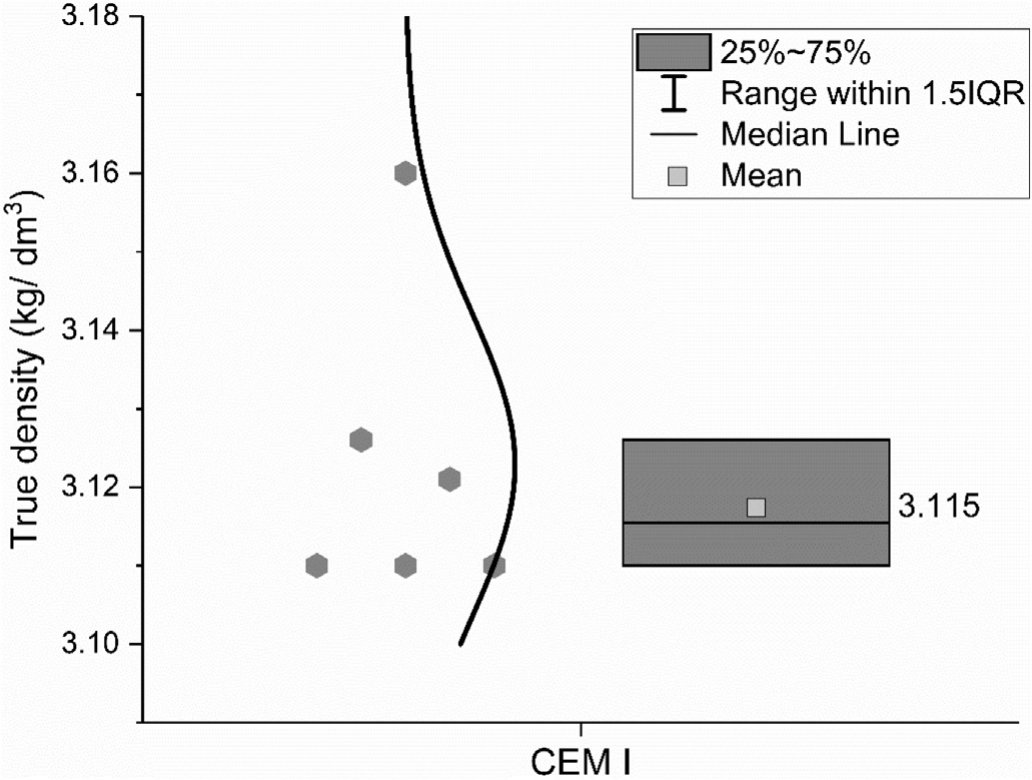
True density of CEM I 42.5 R.

The true density of the CEM I 42.5 R was measured by Helium pycnometer method according to standard EN 1097-7: 2008 [4]. Results are shown in Fig. 4. The same experiment was conducted by different groups, as shown by the hexagon, and then the mean value was calculated.

**Fig. 5:**
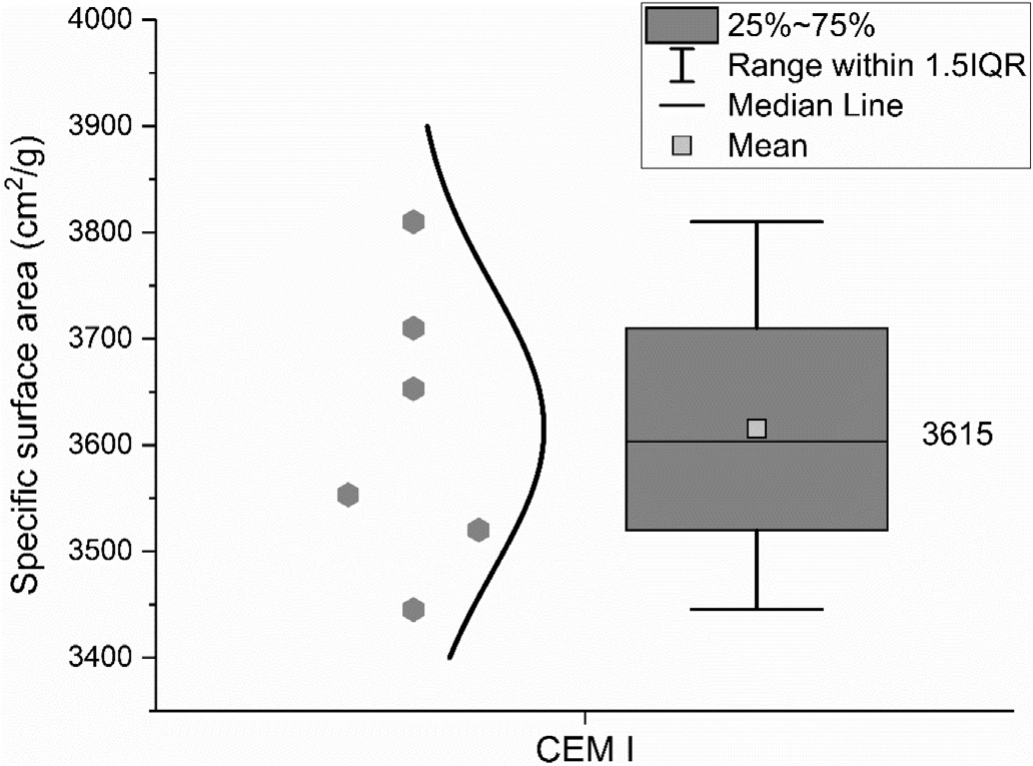
Specific surface area of CEM I 42.5 R measured by the Blaine method.

The specific surface area of the CEM I 42.5 R was measured by the Blaine method according to EN 196-6: 2018 [5] and the results are shown in Fig. 5.

**Fig. 6:**
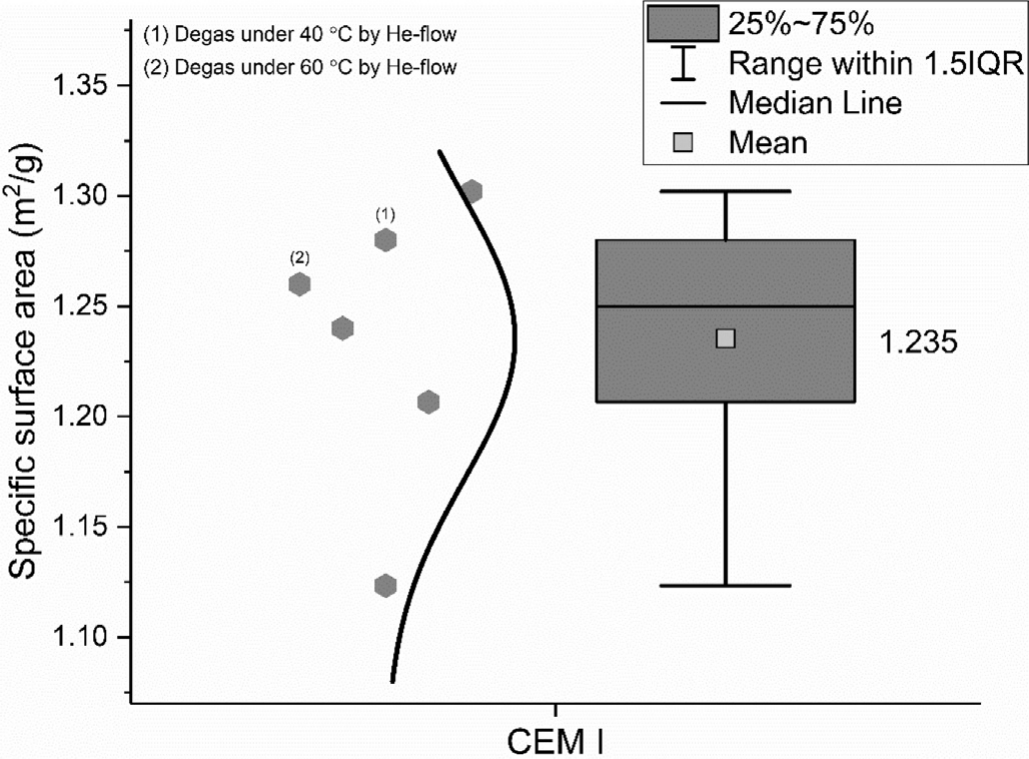
Specific surface area of CEM I 42.5 R measured by the BET method.

The specific surface area of the CEM I 42.5 R was measured by the BET method according to ISO 9277: 2010 [6]. Results are shown in Fig. 6. The numbers in brackets indicate the values from the same sample but different pre-treatment methods that were conducted by the same group.

**Fig. 7:**
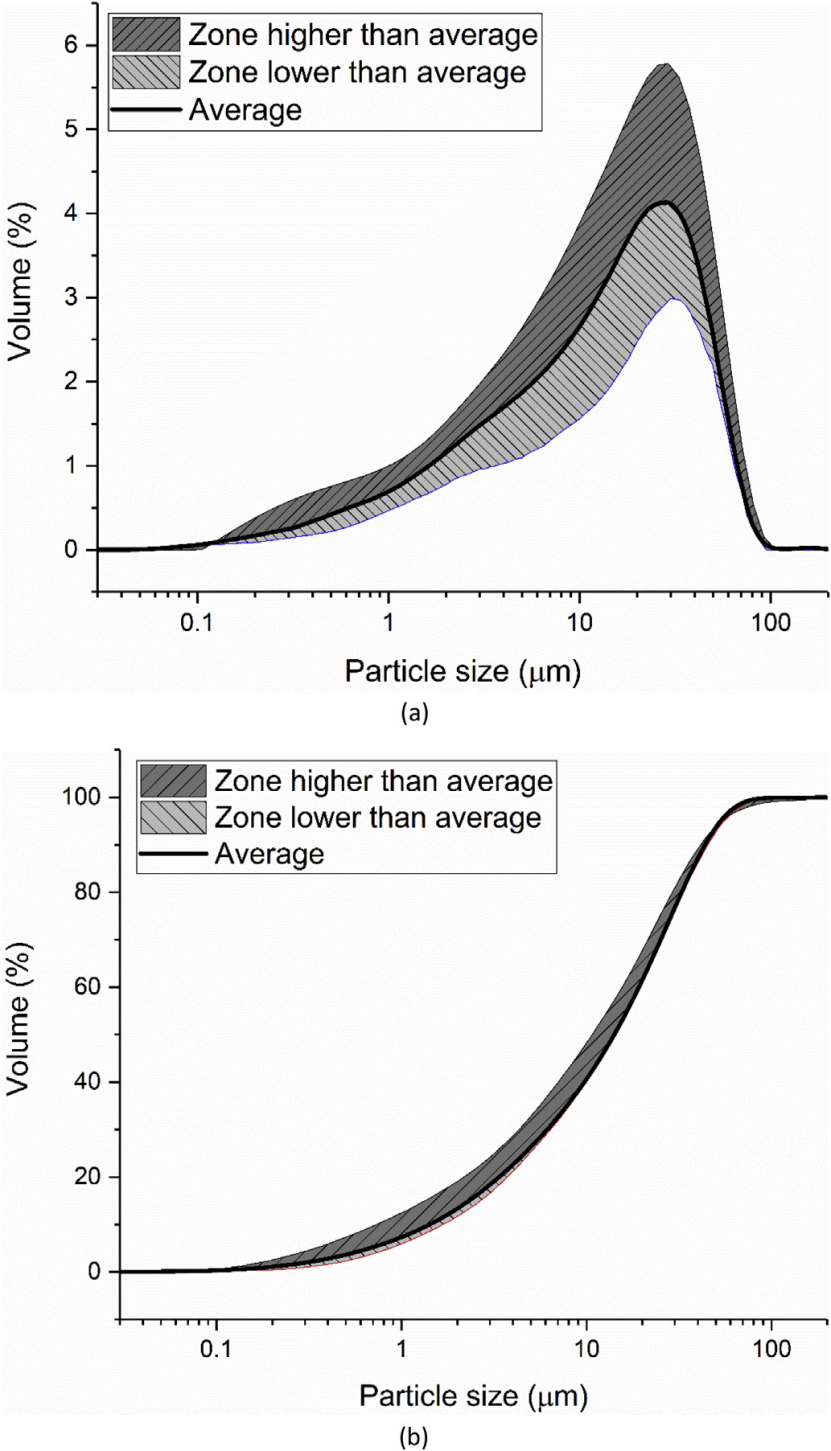
Particle size and distribution of CEM I 42.5 R measured by laser diffraction method; (a) differential curve; (b) Integration curve.

**Fig. 8:**
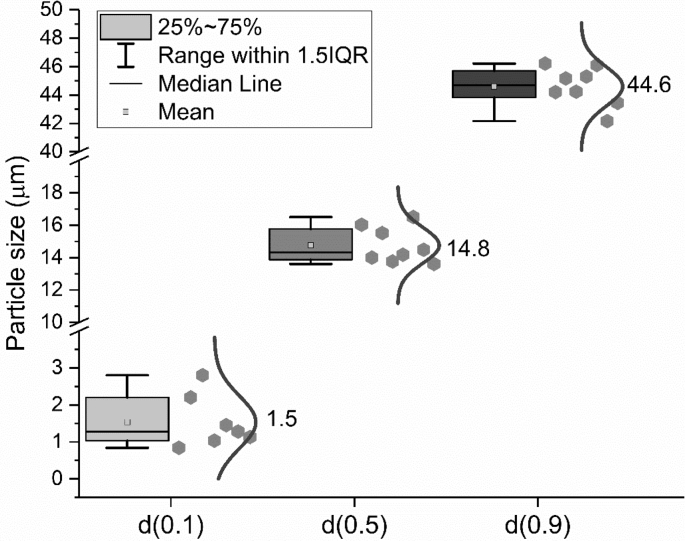
Particle size distribution of CEM I 42.5 R at d (0.1), d (0.5) and d (0.9).

Laser diffraction was applied to measure the particle size distribution (PSD) of the cement by eight different groups according to the method described in ISO 13320: 2009 [7]. The average distribution line was calculated, as shown in Fig. 7. The shadow areas below and above this average line indicate the scope of the testing results. The characterized particle size distributions of the cement (d (0.1), d (0.5) and d (0.9)) are shown in Fig. 8.

### 1.3 Characterization data of other properties

**Fig. 9:**
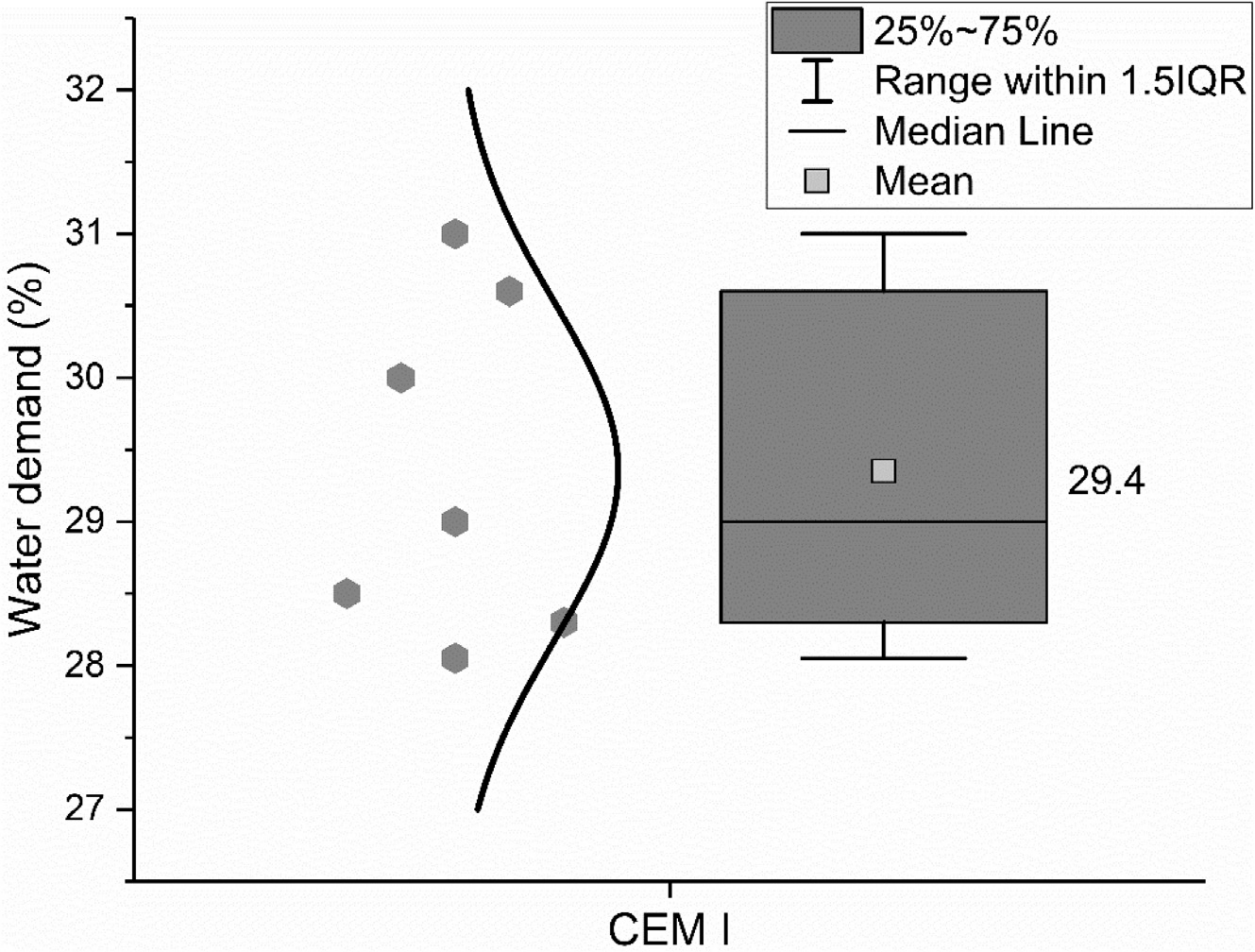
Water demand of CEM I 42.5 R.

**Fig. 10:**
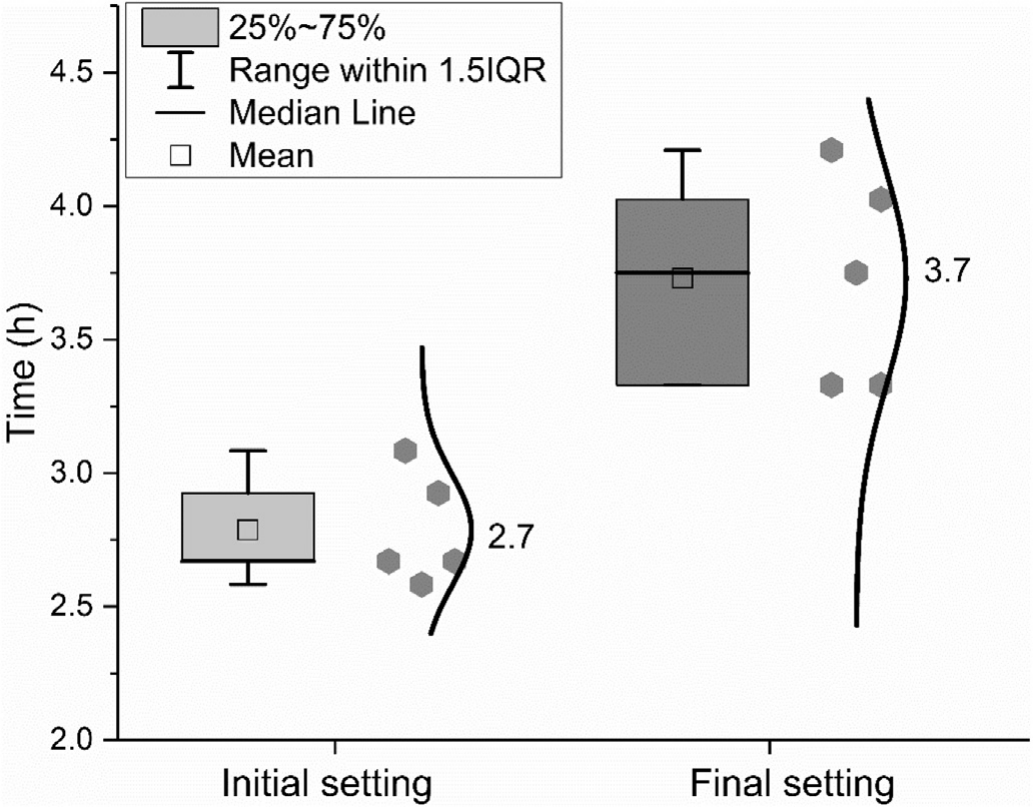
Initial and final setting time of CEM I 42.5 R.

**Fig. 11:**
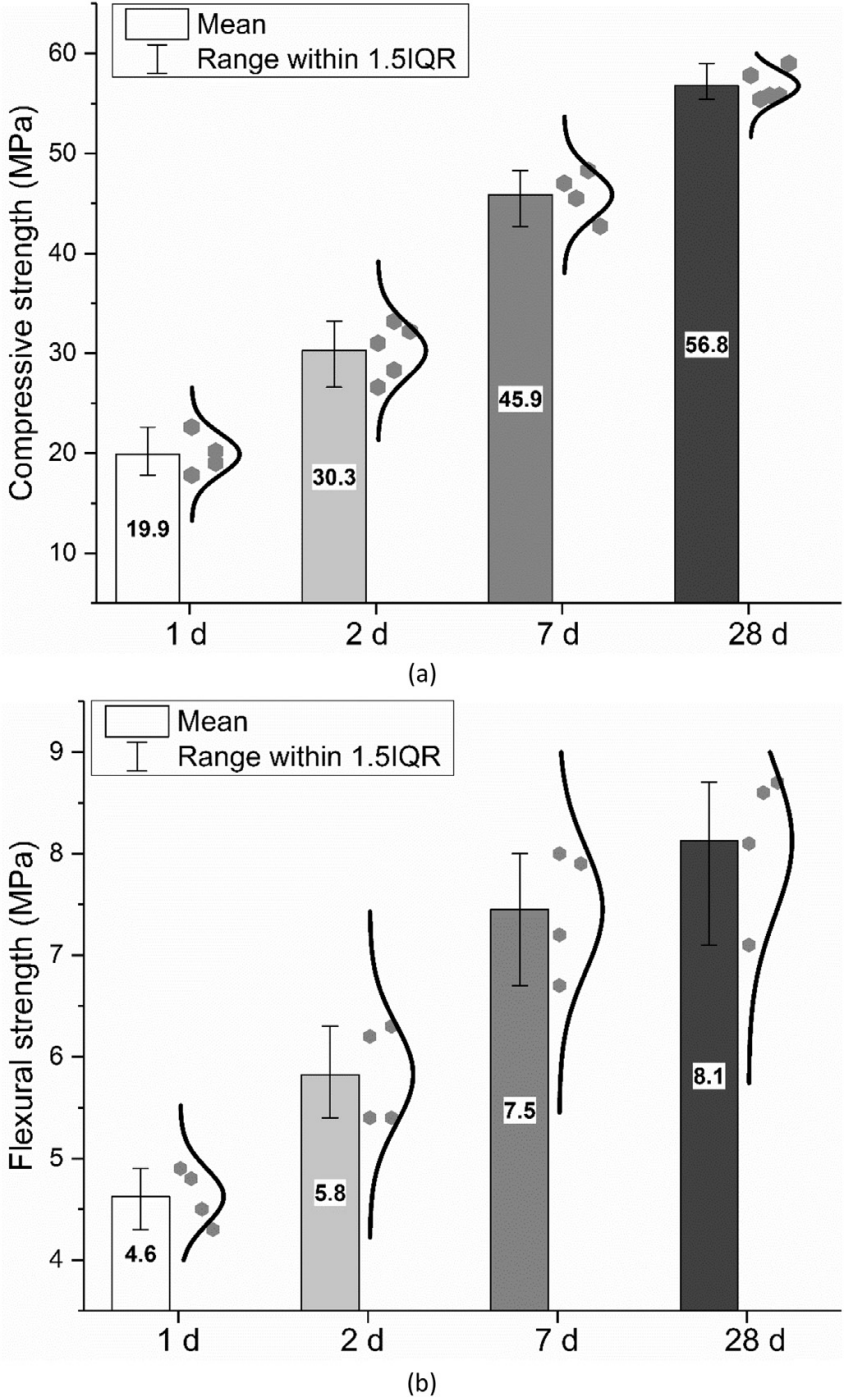
Mechanical strength of hardened cement mortars after curing for certain time; (a) Compressive strength; (b) Flexural strength.

Water demand, as well as initial and final setting time were measured according to the standard EN 196-3: 2016 [8]. Flexural and compressive strength were measured according to the standard EN 196-1: 2016 [9]. The results are shown in Fig. 9, Fig. 10, Fig. 11.

**Fig. 12:**
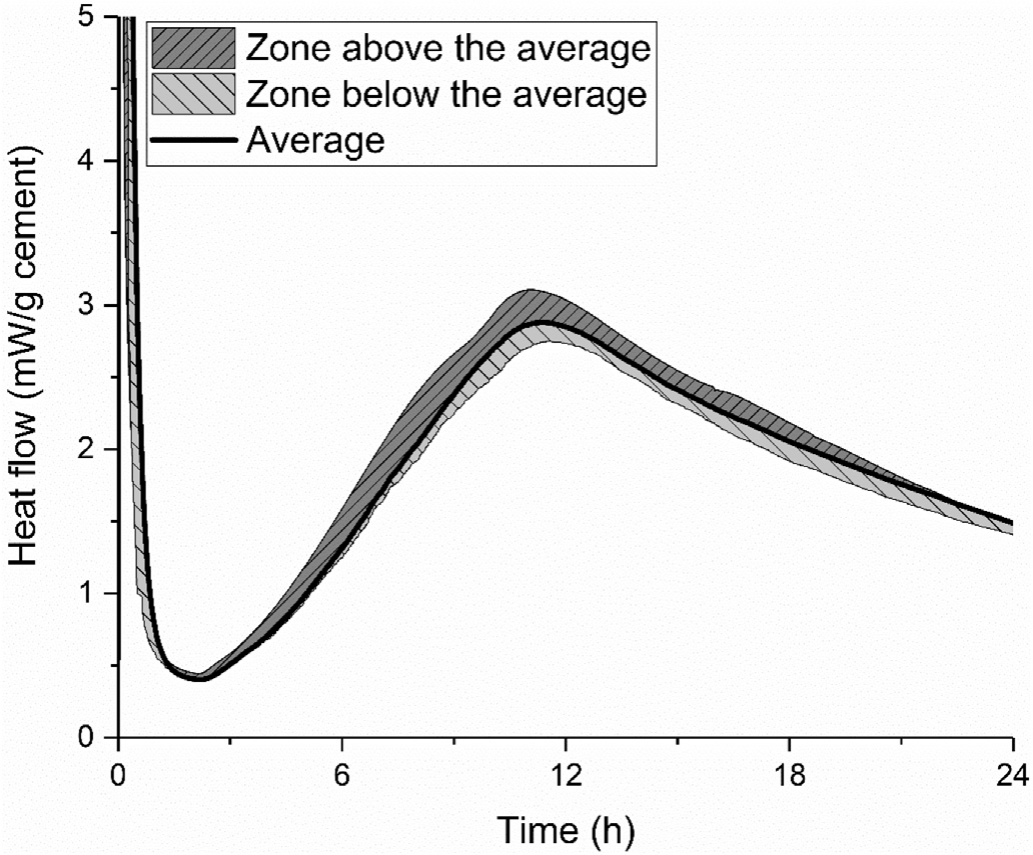
Calorimetry curve of cement paste with water to cement ratio of 0.434 at the temperature of 20 °C.

The cement hydration with a water to cement ratio of 0.434 at the temperature of 20 °C was characterized independently by three different groups according to the method described in EN 196-11: 2018 [10]. The results are shown in Fig. 12. The shadow areas below and above the average line indicate the scope of the test results.

## 2 Experimental design, materials, and methods

All samples analyzed in this campaign stemmed from the same batch of cement production. The sample amount delivered to the different research groups were between a few kilograms up to several tons. The material was stored in closed containers, and the various groups took a representative sample from their own sub-batch.

For the characterizations of the CEM I 42.5 R, EN 196-2: 2013 was applied for the assessment of the oxide composition, insoluble residue and loss on ignition. Density was measured according to EN 1097-7: 2008; specific surface area by the Blaine method was measured according to EN 196-6: 2018 and by BET based on ISO 9277: 2010. Water demand and setting times were tested based on EN 196-3: 2016; flexural and compressive strength were obtained following EN 196-1: 2016. Isothermal heat flow calorimetry was measured according to EN 196-11: 2018. Particle size distribution was evaluated based on ISO 13320: 2009. For the other characterization methods of the CEM I 42.5 R, the specific experiment design and methods are explicated below.

SEM images were recorded on uncoated cement powder with a Nova NanoSEM 230 (FEI, Netherlands) equipped with a field-emission gun (Schottky emitter). For lower magnification, a low-vacuum-detector (LVD) applying 12 kV acceleration voltage and 0.9 mbar was used. For higher magnification, a through the lens detector (TLD) at 2 kV and 22 pA electric current was used under high vacuum conditions.

For the characterization of phase contents, powder-XRD combined with quantification of the patterns was used. In different research groups, different XRD devices with different analysis software were used. In one research group, XRD was performed in a Siemens D5000 with operation parameters given elsewhere [11]. Rietveld refinement was performed with the software Profex (3.12.1). In the software package, the fundamental parameters approach for Rietveld refinement was applied [12]. In another research group, the software package of Bruker Topas V5.0 was used for Rietveld refinement. In the software package, the fundamental parameters approach for Rietveld refinement was implemented [13]. Additionally, an external standard [14] was applied in order to estimate the amorphous content of the CEM I 42.5 R, which was found to be negligible.

## Figures and Tables

**Table 2:** Oxide composition of CEM I 42.5 R and the corresponding standard deviation.

	CaO	SiO_2_	Al_2_O_3_	Fe_2_O_3_	MgO	K_2_O	Na_2_O	TiO_2_	P_2_O_5_	Mn_2_O_3_	SO_3_^a^	SO_3_^b^	LOI	Cl^−^	Insoluble residue	Sum
Composition (wt.-%)	64.4	20.4	5.4	2.6	1.4	0.77	0.22	0.29	0.14	0.07	2.7	3.11	1.87	0.02	1.04	100.12
Standard deviation	0.85	0.16	0.19	0.21	0.15	0.09	0.01	0.02	0.04	0.02	0.35	0.24	0.05	0.003	0.12	0.25

a Measured by XRF.

b Analysis by other methods.

**Table 3:** Phase contents of CEM I 42.5 R and the corresponding standard deviation.

	C_3_S	C_2_S	C_3_A (orth.)	C_3_A (cub.)	C_4_AF	Anhydrite	Bassanite	Arcanite	Calcite	Quartz	Periclase	Sum
Composition (wt.-%)	55.8	14.6	3.6	7.3	7.4	2.2	2.7	0.5	3.7	0.9	0.4	99.5
Standard deviation	1.79	0.45	0.58	0.50	0.97	0.27	0.45	0.23	0.19	0.21	0.11	0.50

**Table 4:** Physical properties of CEM I 42.5 R and the corresponding standard deviation.

	Density (kg/dm^3^)	Specific surface area^a^ (cm^2^/g)	Specific surface area^b^ (m^2^/g)	Particle size (μm)		
				d (0.1)	d (0.5)	d (0.9)
Average value	3.115	3615	1.235	1.5	14.8	44.6
Standard deviation	0.0068	122.6	0.0584	0.66	1.03	1.29

a Measured by Blaine method.

b Measured by BET method.

**Table 5:** Other properties of CEM I 42.5 R and the corresponding standard deviation.

	Water demand (wt.-%)	Setting time (h)		Compresive strength (MPa)				Flexural strength (MPa)			
		Initial	Final	1 d	2 d	7 d	28 d	1 d	2 d	7 d	28 d
Average value	29.4	2.7	3.7	19.9	30.3	45.9	56.8	4.6	5.8	7.5	8.1
Standard deviation	1.09	0.19	0.36	1.77	2.46	2.08	1.40	0.24	0.43	0.53	0.63
